# Identification of Differentially Expressed Micrornas Associate with Glucose Metabolism in Different Organs of Blunt Snout Bream (*Megalobrama amblycephala*)

**DOI:** 10.3390/ijms18061161

**Published:** 2017-05-31

**Authors:** Ling-Hong Miao, Yan Lin, Wen-Jing Pan, Xin Huang, Xian-Ping Ge, Ming-Chun Ren, Qun-Lan Zhou, Bo Liu

**Affiliations:** 1 Wuxi Fisheries College, Nanjing Agricultural University, Wuxi 214081, China; miaolh@ffrc.cn (L.-H.M); pan_787856wj@163.com (W.-J.P.); 17798746965@163.com (X.H.); liub@ffrc.cn (B.L.); 2 Key Laboratory of Freshwater Fisheries and Germplasm Resources Utilization, Ministry of Agriculture, Freshwater Fisheries Research Center, Chinese Academy of Fishery Sciences, Wuxi 214081, China; liny@ffrc.cn (Y.L.); renmc@ffrc.cn (M.-C.R.); zhouql@ffrc.cn (Q.-L.Z.)

**Keywords:** microRNAs, wheat starch, glucose and lipid metabolism, *Megalobrama amblycephala*

## Abstract

Blunt snout bream (*Megalobrama amblycephala*) is a widely favored herbivorous fish species and is a frequentlyused fish model for studying the metabolism physiology. This study aimed to provide a comprehensive illustration of the mechanisms of a high-starch diet (HSD) induced lipid metabolic disorder by identifying microRNAs (miRNAs) controlled pathways in glucose and lipid metabolism in fish using high-throughput sequencing technologies. Small RNA libraries derived from intestines, livers, and brains of HSD and normal-starch diet (NSD) treated *M. amblycephala* were sequenced and 79, 124 and 77 differentially expressed miRNAs (DEMs) in intestines, livers, and brains of HSD treated fish were identified, respectively. Bioinformatics analyses showed that these DEMs targeted hundreds of predicted genes were enriched into metabolic pathways and biosynthetic processes, including peroxisome proliferator-activated receptor (PPAR), glycolysis/gluconeogenesis, and insulin signaling pathway. These analyses confirmed that miRNAs play crucial roles in glucose and lipid metabolism related to high wheat starch treatment. These results provide information on further investigation of a DEM-related mechanism dysregulated by a high carbohydrate diet.

## 1. Introduction

Carbohydrates are an important source of energy and organic carbon for living organisms. Through a carbohydrate metabolism process, it could transfer into other carbonaceous compounds, including amino acids, fat, and nucleotides, to maintain the nutrition metabolism in organisms [1]. For majority of the farmed animals, the major energy sources are low molecular weight sugar, starch, and various cell walls and stored non-starch polysaccharides [1]. Moreover, dietary nutrition content determines growth performance, body composition, and the quality of muscle and flavors [2,3,4], and subsequently determines the marketing status of consumer requirements. Therefore, dietary nutrition as well as the feed formula or metabolism regulation of nutritional physiology are always the research hotspots in aquaculture industry.

Blunt snout bream (*Megalobrama amblycephala, M. amblycephala*) is a widely favored freshwater herbivorous fish species due to its delicacy and a high ratio of edible meat weight to body weight. Blunt snout bream is naturally distributed in the Yangtze River, China and are mainly farmed in Chinese freshwater polyculture systems [5,6]. Due to its high economic value and market demand, *M. amblycephala* is becoming a main aquaculture species and its production is rapidly growing [5]. As reported, the output of *M. amblycephala* reached over 796,000 tons in China in 2015 [6,7]. Moreover, the digestive function and disease resistance ability of *M. amblycephala* are highly susceptible to high-fat, high-glucose, and high-protein diets. Research showed that the appropriate carbohydrate ratio in the feed of *M. amblycephala* is 30–34%, and higher carbohydrate feeding results in glucose intolerance responses including declined glucose metabolic enzyme activity and liver antioxidant abilities, persistent hyperglycemia, and even increased susceptibility to pathogenic bacteria [8,9,10]. For molecular illumination, excessive glucose load in *M. amblycephala* caused a consequence of metabolic disorders and dysfunctions in biological processes and molecular pathways in glucose and lipid metabolisms, including the peroxisome proliferator-activated receptor (PPAR) pathway [11,12,13], the cyclic adenosine monophosphate(cAMP) response element-binding protein [14], and the insulin signaling pathway [12,13,15], which associate with metabolism regulation. In addition, this dysregulation could be regulated by abnormal expression of genes and microRNAs (miRNAs, single-stranded non-coding RNA molecules length at 18–25 nucleotides) in response to those stimuli.

Emerged critical regulatory roles of miRNAs in gene expression and biological functions have been reported in various species and disease related mechanisms [16,17,18,19,20]. For instance, recent advances in miRNAs revealed that miR-122 and miR-33 play essential roles in regulation of cholesterol and fatty-acid homeostasis [6,21]. The differentially expressed miRNAs (DEMs) including miR-30c, miR-145, and miR-15a-5p in *M. amblycephala* that responded to a high fat diet had been identified and enriched into various biological process categories and pathways using high throughput sequencing analysis [6]. Moreover, sequencing analysis has been used to identify DEMs in *M. amblycephala* with different growth rates [16].

High carbohydrate diet related diabetes and obesity are associated with differentiated gene expression and promotes weight gain. Glucose metabolism related DEMs have been identified in studies in vivo, including in *Chronic pistachio* taking prediabetes patients [22] and dietary obesity mouse model [23,24]. However, a comprehensive illustration of the mechanisms with the identification of miRNA controlled pathways resulting from high carbohydrate diet feeding is necessary to understand their function in glucose metabolism in fish. Herein, we used high-throughput sequencing technologies to detect and identify the DEMs and associated mechanisms related to high wheat starch diets. In this study, small RNA libraries from intestines, livers, and brains of *M. amblycephala* were established, followed by sequencing, data processing, identification of DEMs, predication and annotation of DEM targets. Hundreds of DEMs and numerous predicated targets were identified. Annotation results showed that those DEMs in intestines, livers, and brains are associated with a broad range of biological processes and pathways. These results in this present study may provide a better understanding of dysregulated miRNAs and biological processes in response to high carbohydrate diets, and may be used to explore glucose and lipid metabolic regulation of *M. amblycephala*.

## 2. Results

### 2.1. Small RNA Profiles in M. amblycephala

**Figure 1:**
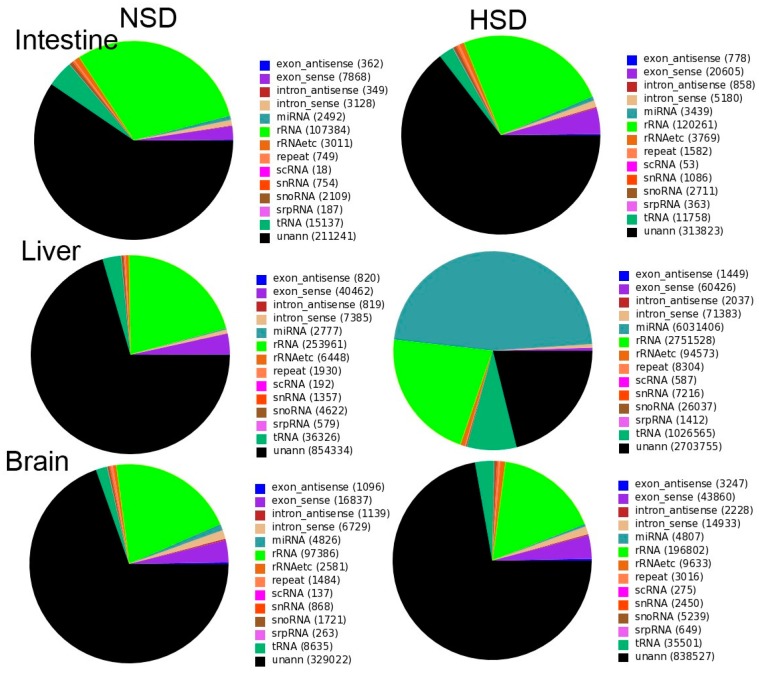
Annotation of total small RNAs derived from Illumina sequencing of *Megalobrama amblycephala* all RNAs libraries. (NSD: the fishes fed with 34% level starch, HSD: the fish fed with 51% level starch).

Independent small RNA libraries were generated from *M. amblycephala* intestines, livers and brains fed with normal starch diet (34%, NSD) and high starch diet (51%, HSD) groups using the Illumina Hiseq 2500 platform (Illumina, San Diego, CA, USA) to identify miRNAs involved in glucose metabolism. A total of 14,607,507, 14,512,592, 17,593,491, 14,313,923, 14,176,105 and 17,261,129 raw reads were generated from the NSD intestine, HSD intestine, NSD liver, HSD liver, NSD brain, and HSD brain libraries, respectively (Supplementary Table S1). After filtering out the poor quality reads (3′ adaptor null reads, reads with 5′ adaptor contaminants, and reads shorter than 18 nt), clean reads reduced to 12,458,528, 11,477,918, 12,934,301, 12,786,678, 12,068,998 and 14,703,085, respectively, which represented 354,789, 486,266, 1,212,012, 712,816, 472,724, and 1,161,167 unique small RNAs (Supplementary Tables S1 and S2). Most of the small RNAs were 21–23 nt in length (Supplementary Figure S1), and few were commonly expressed both in NSD and HSD groups (Supplementary Figure S2). After comparing the small RNA sequences with the National Center for Biotechnology Information (NCBI) GenBank and Rfam database, rRNA, tRNA, snRNA, snoRNA, scRNA, repeat DNA, exon antisense, exon sense, intron antisense and intron sense sequences were annotated. These results suggest that *M. amblycephala* intestines, livers, and brains contain large numbers of non-coding small RNAs (Supplementary Figure S3), which may contribute to the regulation of gene expression. The remaining clean reads, including 1,175,102, 2,153,358, 3,457,970, 2,703,755, 2,116,039 and 3,405,194 reads in NSD intestines, HSD intestines, NSD livers, HSD livers, NSD brains, and HSD brain libraries, respectively, consisted of 211,241, 313,823, 854,334, 467,104, 329,022 and 838,527 unique sequences (Figure 1), respectively, were retained for the next analysis.

The selected small RNA sequences were mapped to the genome sequence of grass carp (*Ctenopharyngodon idellus*), which is evolutionarily close to *M. amblycephala,* because there is a lack of genome information for *M. amblycephala*. We chose no tolerance of mismatch for mapping for the selection of the computing algorithm [16]. Subsequently, 10,633,361 reads (85.35%), 9,100,987 reads (79.29%), 8,650,619 reads (66.88%), 9,629,114 reads (75.31%), 9,921,195 reads (82.20%) and 10,621,670 reads (72.24%) in NSD intestines, HSD intestines, NSD livers, HSD livers, NSD brains, and HSD brain libraries, respectively, which represented 100,970 (28.5%), 125,982 (25.91%), 187,046 (15.43%), 172,035 (24.31%), 118,618 (25.09%), and 232,023 (19.98%) unique sRNAs were aligned (Supplementary Table S3). Small RNAs from the same *M. amblycephala* tissues have similar distribution patterns in grass carp chromosomes (Supplementary Figure S4).

### 2.2. Identification of Known miRNAs

To identify known miRNAs, the sequences of the small RNAs in the libraries of the *M. amblycephala* were compared with those of the 316 precursor miRNAs and 420 mature miRNAs of zebrafish and carp from miRBase Version 21.0 (http://www.mirbase.org/) with no more than two mismatches. This analysis resulted in 321, 337, 330, 329, 349 and 348 known miRNAs in the NSD intestines, HSD intestines, NSD livers, HSD livers, NSD brains, and HSD brain libraries, respectively, including a total of 367 mature miRNAs (Supplementary Tables S3 and S4). Moreover, the fact that more than 85% clean reads of our sRNA libraries were matched to known miRNAs confirmed the high enrichment with mature miRNAs. The fact that these miRNAs reads ranged from 1 to 2,712,658 revealed that both the highly- and weakly-expressed miRNAs were identified by Illumina sequencing. The top 20 most abundant miRNAs identified in the NSD intestines, HSD intestines, NSD livers, HSD livers, NSD brains, and HSD brain libraries, respectively, accounted for 87.56%, 83.25%, 72.82%, 80.70%, 72.78% and 73.14% of the total reads mapped to miRBase (Supplementary Table S4). Among these known miRNAs, such as mam-miR-192, mam-miR-143b, mam-miR-26a-5p, mam-miR-128 and mam-miR-100-5p, showed a relatively higher number of sequencing reads in NSD intestines, HSD intestines, NSD livers, and HSD liver libraries.

Additionally, the analysis of base bias on each position of all identified miRNAs was performed. The identified sequences of known miRNAs from six libraries showed a strong bias for U in the first and the 24th nucleotide (Supplementary Figure S5).

### 2.3. Identification of Novel miRNAs

Next, small RNAs from the six *M. amblycephala* libraries were mapped to the grass carp reference genome, but unknown miRNAs were analyzed to identify the unannotated miRNAs. MIREAP software (https://sourceforge.net/projects/mireap/) was used to predict novel miRNAs based on secondary structure, dicer enzyme cleavage sites, and the minimum free energy (MFE) using the MIREAP criteria[16]. Accordingly, we finally obtained 188 putative novel miRNAs in *M. amblycephala* sequencing reads, including 50, 61,57, 81, 126 and 108 novel miRNAs, and 52, 63, 58, 85, 135 and 123 pre-miRNAs, respectively, in the NSD intestines, HSD intestines, NSD livers, HSD livers, NSD brains, and HSD brain libraries (Supplementary Table S5). The novel miRNAs identified from the six libraries had a strong bias for U in the first nucleotide and for G in the 24thnucleotide, respectively (Supplementary Figure S6).

### 2.4. Identification of DEMs

**Figure 2:**
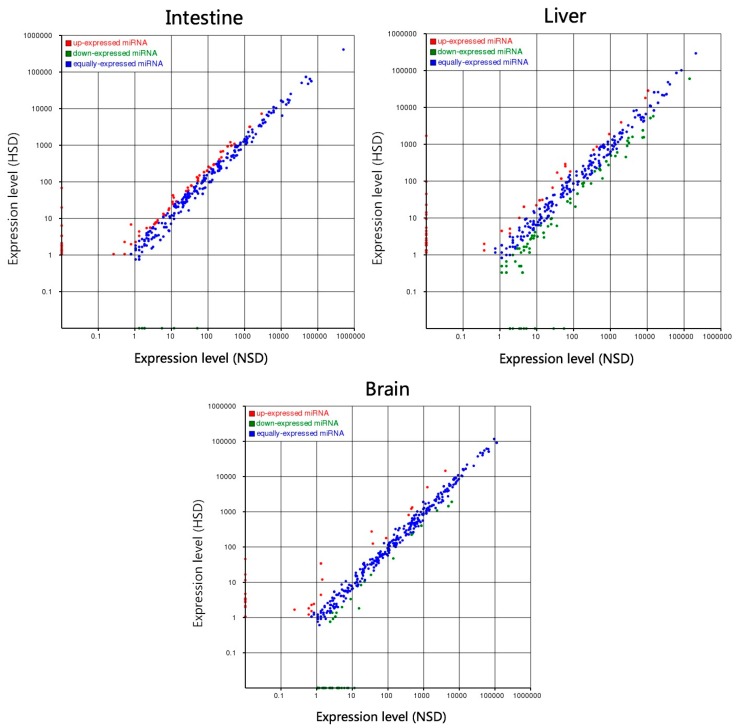
Differentially expressed miRNAs *M. amblycephala.* Red and green represents up- and downregulated miRNAs in high starch diet (HSD) group, compared with low starch diet (NSD) group. |log2FC(Fold change)| ≥ 1 and *p*-value ≤ 0.05.

**Figure 3:**
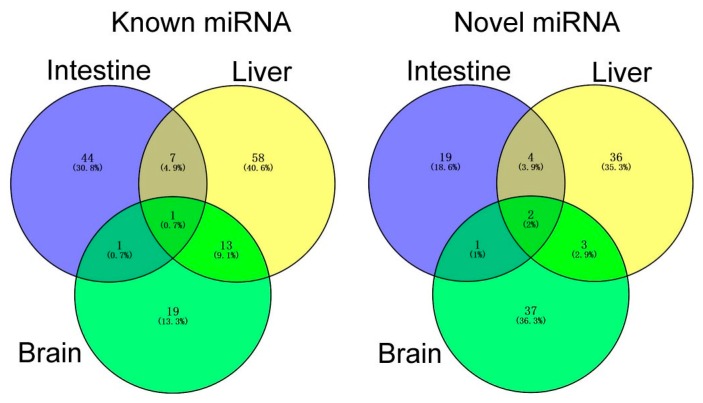
Venn chart of differentially expressed miRNAs *M. amblycephala.*

After removing the sequencing tags fewer than five reads, a total of 79, 124 and 77 miRNAs were identified as DEMs in intestines, livers, and brains between NSD and HSD groups (Figure 2), following the criteria of|log2FC(Fold change)| ≥ 1 and *p*-value ≤ 0.05. Among them, there were 53, 79 and 34 conserved known miRNAs, respectively, plus with 26, 45 and 43 novel miRNAs (Supplementary Table S6). There were 7, 79 and 47 upregulated miRNAs and 72, 45 and 30 downregulated miRNAs in intestines, livers, and brains in the HSD group, in comparison with NSD (Supplementary Table S6). Among those DEMs, only one known DEM (mam-miR-205-5p) and two novel DEMs (mam-miR-n28, and mam-miR-n121) were the common DEM among three organs (Figure 3, other common DEMs, see Supplementary Table S6). However, mam-miR-205-5p was significantly upregulated in HSD intestines, whereas it was significantly downregulated in HSD livers and brains, in comparison with corresponding organs in NSD groups (Supplementary Table S6).

**Figure 4:**
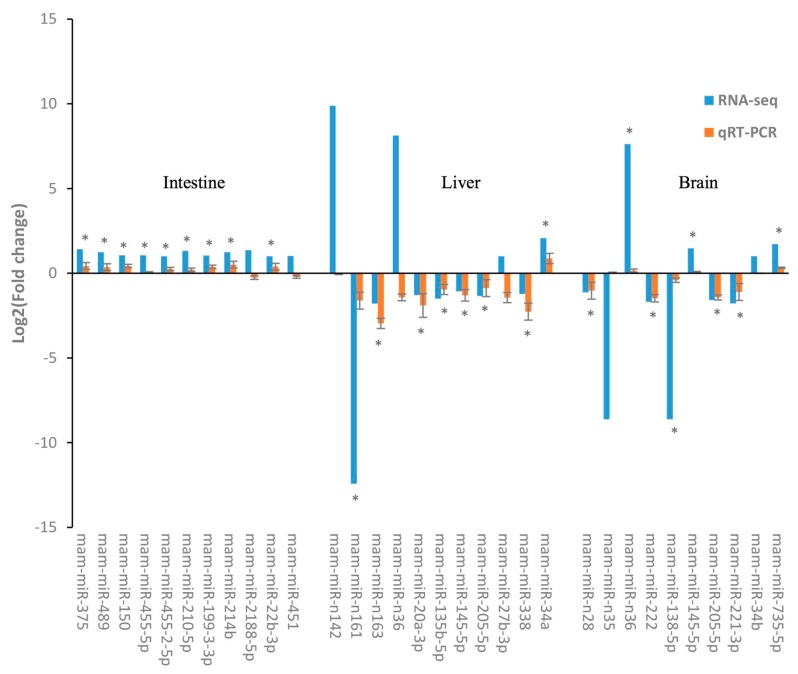
Quantitative real-time PCR (qPCR) analysis for 24 differentially expressed miRNAs in different tissues identified by small RNA sequencing. The stars mean that the results of RNA-seq and qRT-PCR were in consistent and all significant. The error bars were the standard deviation.

Using the clustering analysis, we obtained the expression profiles of these DEMs in samples (Supplementary Figure S7). Using the qRT-PCR, we detected the expression patterns of 32 DEMs randomly selected from the above DEMs and confirmed the high accordance of qRT-PCR results with that of RNA-Seq analysis. These DEMs might be tightly related to the glucose or lipid metabolism in response to HSD treatment. Intriguingly, for all seven of the differential expressed novel DEMs identified by small RNA sequencing analysis, obvious higher log2 (Fold change) values were detected using qRT-PCR in comparison with RNA-seq (Figure 4). Additionally, most of these novel miRNAs in *M. amblycephala* tissues showed more significant differences in relative expression levels by qRT-PCR than those of the conserved miRNAs, such as mam-miR-n142, mam-miR-n161, mam-miR-n35, and mam-miR-n36. Expression patterns of 9 out of 11, 8 out of 11, and 8 out of 10 DEMs, respectively, in intestines, livers, and brains were consistent with the result of RNA-Seq analysis (Figure 4, and the corresponding Supplementary Table S7). We demonstrate that the sequencing data and the qRT-PCR results in this present study are in accordance, and the analyses for DEMs are significative.

### 2.5. Prediction of Potential Targets of DEMs

For the identification of DEM targets, we subjected the 79, 124 and 77 DEMs to miRanda, TargetScan and RNAhybrid at the same time to search for the common targets following the criteria, respectively. Accordingly, 48,334, 48,420 and 48,336 unique targets were predicted for the identified 79, 124 and 77 significant DEMs responded to HSD stimuli (Supplementary Table S8). The results showed that each miRNA targeted numerous unique genes, and one unique gene could be regulated by more than one miRNA. For example, mam-miR-205-5p targets to 368 unique genes and one of the targets of DEM mam-miR-192, unigene7630_LT, also is a target of mam-miR-27b-3p, mam-miR-301c-3p, mam-miR-210-3p, mam-miR-210, and a novel miRNA mam-miR-n134.

### 2.6. Function Analysis of DEM Target Genes

Next, we annotated the biofunction or biochemical signal pathways related to those predicated miRNA targets by subjecting those target unique genes to Gene Ontology database: (available online: http://www.geneontology.org/) and the Kyoto Encyclopedia of Genes and Genomes (KEGG) pathway database (http://www.kegg.jp/). Based on the Gene Ontology (GO) functional annotation, we found these predicted target genes in intestines, livers, and brains were classified into classes of molecular functions, cellular components and biological processes over decades(*p* < 0.05), such as gluconeogenesis (GO:0006094), hexose biosynthetic process (GO:0019319), monosaccharide biosynthetic process (GO:0046364), regulation of macromolecule metabolic process (GO:0060255), glucose metabolic process (GO:0006006),response to carbohydrates (GO:0009743), phosphoenolpyruvate carboxykinase activity (GO:0004611) and carboxy-lyase activity (GO:0016831, Supplementary Table S9). For instance, predicted DEM targets such as glycerol-3-phosphate dehydrogenase 2 (GPD2), phosphoglucomutase 1 (PGM1), and phosphoenolpyruvate carboxykinase 1 (PCK1) enriches into GO terms including gluconeogenesis and the glucose metabolic process.

**Figure 5:**
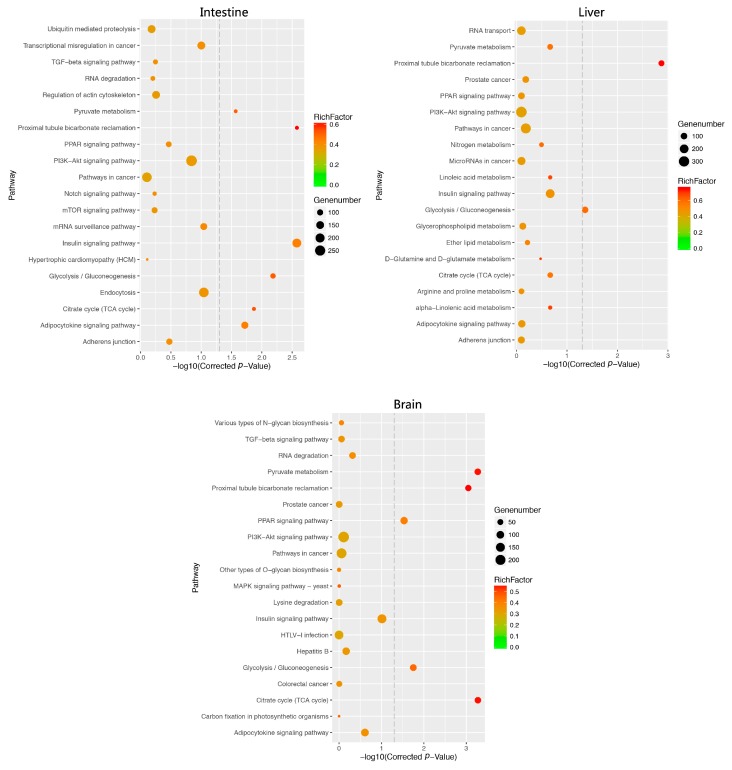
Kyoto Encyclopedia of Genes and Genomes (KEGG) pathway classifications for predicted targets of different expression miRNAs in *M.amblycephala*.

For the KEGG pathway enrichment analysis, we found those predicted target genes associated with numerous glucose/lipid metabolism-related signaling pathways, including pyruvate metabolism (ko00620, mam-miR-20a), citrate cycle (tricarboxylic acid cycle, ko00020), glycolysis/gluconeogenesis (ko00010), PPAR signaling pathway (ko03320), insulin signaling pathway (ko04910), adipocytokine signaling pathway (ko04920), and PI3K-Akt signaling pathway (ko04151, Figure 5, Supplementary Tables S10 and S11). Accordingly, these DEMs that are significantly involved in GO and KEGG terms of glucose and lipid metabolisms, including mam-miR-34a, mam-miR-205-5p, mam-miR-735-5p, and mam-miR-n142, were identified (Supplementary Table S11). Among these miRNA targets, EP300A, PCK1, Akt1, insulin receptor substrate 4 (IRS4), raf-1 proto-oncogene, serine/threonine kinase (Raf1), and glycogen synthase 1 (GYS1) are important factors related to glucose metabolisms, including glycogen synthase, storage, insulin desensitization and resistance. Other targets including PLA2G7 were enriched into glycerophospholipid metabolism (ko00564) and ether lipid metabolism (ko00565) pathways.

### 2.7. PPI (Protein–Protein Internetwork) and Interaction Analysis

**Figure 6:**
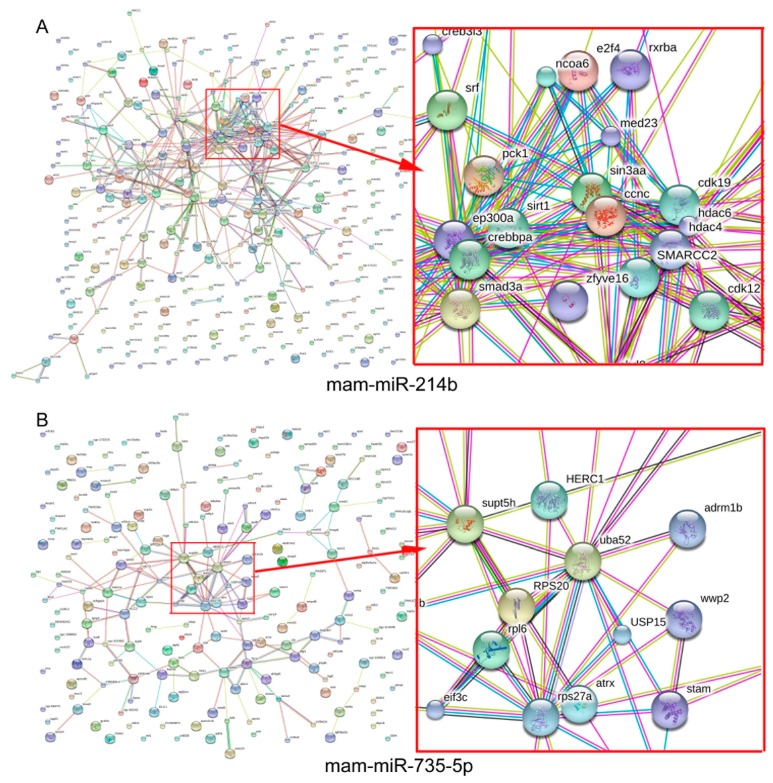
Protein–protein interaction (PPI) networks for the predicted targets of differentially expressed mam-miR-214b and mam-miR-735-5p in *M. amblycephala*. Network nodes represent proteins, and edges represent protein–protein associations.

Interaction analyses of gene products do better at understanding interactions between DEM targets. To explore the interaction between predicted targets, the PPI networks of several DEM targets were predicted using a Search Tool for the Retrieval of Interacting Genes/Proteins (STRING) database. We constructed several PPIs including two generated from interactions between mam-miR-735-5p and mam-miR-214b predicated targets, respectively (Figure 6, more PPIs of four DEMs are available in Supplementary Figure S8). In PPI networks, we observe that the target genes interact with each other (Figure 6, lines between nodes indicates interactions). For example, PCK1 interacts with other predicated targets of mam-miR-214b, including SRT1, CDK19, Sin3aa, EP300A, SMARCC2 and SMAD3a.

The interactions between targets of mam-miR-735-5p, a specifically upregulated miRNA in brains by HSD, were also predicted and visualized (Figure 6B). Direct interaction between ribosomal protein S (RPS) 27a (ubiquitin fusion gene Uba80), Uba52, ribosomal protein L (RPL) 6 and other gene products (nodes) are available in Figure 6B.

## 3. Discussion

Blunt snout bream is a widely favored freshwater herbivorous fish species, and its flavoris highly susceptible to feeding prescription design. Despite the reduced taste, higher carbohydrate feeding prescription design (>34%) might result into glucose intolerance responses in blunt snout bream [8,9,10]. Using Illumina sequencing and bioinformatics analysis, we identified 79, 124 and 77 DEMs (including 53, 79 and 34 conserved known miRNAs, respectively) that responded in total to HSD treatment in intestines, livers, and brains of *M. amblycephala*, respectively. GO classification and KEGG pathway analysis for predicted targets of the DEMs showed that these DEMs were associated with the PPAR pathway [11,12,13], the cAMP response element-binding protein [14], and the insulin signaling pathway [12,13,15], which are associated with metabolism regulation of glucose and lipid metabolic pathways and biosynthetic processes, demonstrating the important roles of these DEMs in glucose and lipid metabolisms.

miRNAs play crucial roles in regulation of biological functions through modulating gene expression [16]. For example, Zhang et al. identified DEMs, including miR-30c and miR-145 in *M. amblycephala* that responded to high fat diet throughput sequencing analysis [6]. Among the identified known DEMs, miRNAs including mam-miR-192, mam-miR-128 and mam-miR-100-5p showed a relatively higher number of sequencing reads in NSD intestines, HSD intestines, NSD livers, and HSD liver libraries (Supplementary Table S4), and they had been identified to be associated with immune function and basic physical development [25,26,27,28]. In this present study, we demonstrated that mam-miR-205-5p was significantly upregulated in HSD intestines in comparison with NSD intestines (Supplementary Table S6). In addition, previous reports showed that dysregulation of miR-205-5p was involved in the cancers, cholesterol metabolism, and steroid genesis [29,30,31]. Moreover, we detected the expression patterns of identified DEMs in response to HSD treatment using qRT-PCR and confirmed the accordant relative expression levels of these DEMs. Taken together, we revealed that the abnormal expression of mam-miR-192, mam-miR-128, miR-205-5p, and mam-miR-100-5p identified by Illumina sequencing were in response to and took important roles in starch-associated metabolism.

The identification of miRNA targets is a crucial and necessary step to understand the regulatory functions of miRNAs. We subjected the 79, 124 and 77 DEMs to miRanda [32], TargetScan [33] and RNAhybrid [34] and found that one miRNA might target to numerous unique genes, suggesting the strong seed complementarity and evolutionary conservation in the complementary targets region. Biofunction annotation showed that these predicated targets of DEMs in intestines, livers, and brains, such as GPD2, PGM1, PCK1, EP300A, PLA2G7, and IRS4, were significantly classified into categories of glycolysis/gluconeogenesis, glucose metabolic process, phosphoenolpyruvate carboxykinase activity, citrate cycle, adipocytokine signaling pathway, and insulin signaling pathway (Supplementary Tables S9–S11). Those targets of EP300A, PCK1, Akt1, IRS4, Raf, and GYS1 are important factors relating to glucose metabolisms, including glycogen synthase, storage [35,36,37], insulin desensitization and resistance [38,39,40]. Targets of GPD2, PGM1 and PCK1 also took important roles in glucose and lipid metabolism by modulating functions such as insulin resistance and glyceroneogenesis [37,41,42]. PLA2G7, a potent pro- and anti-inflammatory molecule that encodes lipoprotein-associated phospholipase A-2 and has been implicated in multiple inflammatory disease processes [43,44], was one of the predicated targets of mam-miR-n161 and was enriched into glycerophospholipid metabolism (ko00564) and ether lipid metabolism (ko00565) pathways. The fact that all of these factors enrich into more than one pathway including insulin signaling pathway, PI3K-Akt signaling pathway, PPAR signaling pathway, and adipocytokine signaling pathway, confirms that DEMs are related to the glucose and lipid metabolism by regulating those important signaling pathways via their targets.

To understand the interaction between miRNAs, we predicted the interactions in the PPI networks using a STRING database. Four PPI networks including mam-miR-735-5p and mam-miR-214b predicated targets (Figure 6 and Supplementary Figure S8) were constructed just for instance. Among the targets, we found that PCK1 interacted with other predicated targets of mam-miR-214b including SRT1, CDK19, and EP300A. As reported, PCK1 is repressed by glucose administration and expressed in glucose absence [45]. PCK1 is a candidate diabetes and obesity gene and the mutations at PCK1 locus could affect the expression of PCK1 in adipose tissues [37]. Another mam-miR-214b target SIRT1, whose loss relates to glucose-stimulated insulin secretion and activation improves insulin sensitivity of type 2 diabetes [46,47], has been reported to be protective against high-fat diet-induced metabolic damage and thus may be explored as a therapeutic target for type 2 diabetes treatment [47,48]. In addition, GO and KEGG analyses show PCK1 enriched into biological function terms including glycolysis/gluconeogenesis and insulin signaling pathway (Supplementary Tables S9 and S10).

Previous bioinformatics analysis showed some of the targets of mam-miR-735-5p including RPS27a and Uba52 were involved in the PPAR signaling pathway in KEGG pathway enrichment analysis (Supplementary Table S10). As reported, the Uba80 encodes ubiquitin fused to ribosomal protein S27a, which promotes proliferation, inhibits cell apoptosis, and responses to ribosomal stress [49,50,51]. PPAR-α and PPAR-γ agonist, a stress-induced transcription factor in response to reducing oxidative stress and others, benefit brain ischemia and injury recovery by protecting against neuron apoptosis [52,53]. Moreover, brain PPAR-γ contributes to obesity and insulin sensitivity [54], and the interaction of ubiquitin and PPAR pathway provides a new partner for controlling insulin signaling [55,56,57,58]. These results suggest that mam-miR-735-5p play a crucial role in the insulin signaling pathway by participating in the PPAR pathway through its targets such as RPS27a. In addition, we conclude that HSD treatment for *M. amblycephala* affects biological functions associated with pathways including insulin and PPAR pathway, which associates with glucose and lipid metabolism.

## 4. Materials and Methods

### 4.1. Fish and Experimental Procedures

All experimental protocols and feeding scheme were approved by the Bioethical Committee of Freshwater Fisheries Research Center (FFRC) of Chinese Academy of Fishery Sciences (CAFS) (BC 2013863, 9/2013). One hundred and twenty healthy fish were provided by the Nanquan aquaculture base of the FFRC. All fish were allowed to acclimatize for 15 days before experiment. After acclimatization, fish were placed in a 480 L tank supplemented with recirculating aquaculture system under a 12D/12L light cycle at 28 °C. Fish (mean weight 14.82 ± 0.22 g) were randomly divided into two groups, 20 fish for one tank, according to the feeding scheme: the normal control group (Control) fed with a normal starch diet (34% starch, NSD, *n* = 60), and the experimental group fed with a high starch diet (51% starch, HSD, *n* = 60). Fish were hand-fed to apparent satiation three times a day (6:00 a.m. to 6:30 a.m., 12:00 p.m. to 12:30 p.m., and 6:00 p.m.to 6:30 p.m.) for 8 weeks. At the end of experiment, fish were killed, and intestines, livers, and brains were immediately separated from corpses. Tissues were immediately frozen in liquid nitrogen, stored at −80 °C and prepared for RNA isolation.

### 4.2. Small RNA Library Preparation and Sequencing

Total RNA was isolated from frozen tissues using TRIzol (Invitrogen, Carlsbad, CA, USA) according to the manufacturer’s protocol. RNA samples were digested using DNase I (New England Biolabs, Frankfurt, Germany), and RNA purity was assessed using a Nanodrop2000 spectrophotometer (Thermo Fisher Scientific, BRIMS, Cambridge, MA, USA). Six pools (3 controls for intestines, livers, and brains and 3 corresponding pools for HSD) of RNA samples were generated by pooling samples from 3 fish for each pool. The purified RNA pools were ligated with Illumina 3′ and 5′ adapters (Illumina, San Diego, CA, USA) using T4 ligase (New England Biolabs, Frankfurt, Germany). RNA was reverse-transcribed into the first strand cDNA using reverse transcriptase and amplified by PCR for 15 cycles using primers complementary to the adaptor sequences. Then, nucleotide fractions at 140–150 bp length were purified for Illumina sequencing library preparation. For sequencing, each library was loaded into one lane of a 1 × 50 bp single-end Illunima Hiseq 2500 run (Illumina, San Diego, CA, USA). Sequence data for each experimental group were deposited in the National Center for Biotechnology Information (http://www.ncbi.nlm.nih.gov/) with accession number: PRJNA374616.

### 4.3. Sequencing Analysis for miRNA Profiling

The sequence analysis for miRNA profiling was performed as previously described [6]. Firstly, some sequences were removed for data cleaning, which includes getting rid of poor quality reads, 3′ adaptor null reads, reads with 5’ adaptor contaminants, and reads shorter than 18 nt and so on. The remaining sequences were used for mapped to the whole genome sequence of grass carp (*Ctenopharyngodon idellus*) using the Short Oligonucleotide Analysis Package (SOAP) program (http://soap.genomics.org.cn) with a tolerance of no mismatch [59]. Then, rRNAs, tRNAs, snRNAs and snoRNAs in the matched sequences were filter out by blasting against Rfam (http://rfam.sanger.ac.uk/) and NCBI GenBank (http://www.ncbi.nlm.nih.gov/genbank/) databases. After being classified into different categories based on sequence similarity, the remaining reads were aligned to the miRNA precursor of zebrafish and carp in the miRBase version 21 database (http://www.mirbase.org/) to identify conserved miRNAs [6]. All of the identified candidate novel miRNAs in this study fulfilled the MIREAP (http://sourceforge.net/projects/mireap) criteria [6].

### 4.4. Data Normalization, Processing and Identification of Differential Expressed miRNAs (DEMs)

The quantile normalization of miRNA sequencing data performed according to the previous methods [6,60]. Briefly, sequencing data was normalized using the following formula: normalized expression = (actual miRNA sequencing readscount/total miRNAs reads count) × 1,000,000. The expression value was modified to 0.01 if the normalized expression (NE) value of a given miRNAwere zero. The miRNAs with NE values less than 1 in two corresponding groups were not considered as DEMs and were removed in future analyses. The cut-off criteria for DEMs were |log2FC (Fold change)| ≥ 1 and *p*-value ≤ 0.05 between two fed schemes. Then, the expression patterns of DEMs in experimental groups were analyzed by cluster analysis using Cluster 3.0 and Java Tree View software packages [61].

### 4.5. Target Prediction for DEMs

The target genes of DEMs were identified using the RNAhybrid (http://bibiserv.techfak.uni-bielefeld.de/rnahybrid) [34], TargetScan (http://www.Target scan.org/) [33] and miRanda (http://www.microrna.org/microrna/getGene Form.do) [32] prediction packages. The criteria used for RNAhybrid target prediction were the same as previously described [6]. Targets predication used for TargetScan and miRanda were with default settings. The common targets identified by RNAhybrid, TargetScan and miRanda were considered DEM targets in this study. The target reference sequences were assembly blunt snout bream transcripts that come from previous study.

### 4.6. Enrichment Analysis for DEM Targets

Gene Ontology (GO) analysis is a functional study method for large-scale transcriptomic data or genomic data [62]. The Kyoto Encyclopedia of Genes and Genomes (KEGG) pathway database informs people of how molecules or genes work [63]. In order to investigate the related biofunction of DEM targets in the glucose metabolism, the biological function of the DEM targets was annotated using Gene Ontology database: (http://www.geneontology.org/), and the pathways of biochemical and signal transduction significantly associated with the DEM targets were determined through the KEGG pathway analysis (http://www.kegg.jp/) [64]. A category was considered significantly enriched when its *p*-value < 0.05.

### 4.7. MiRNA-mRNA Regulatory Network and PPI (Protein–Protein Internetwork) and the Module Analysis

To better understand interactions between DEM targets, the PPI network was predicted using a STRING (Search Tool for the Retrieval of Interacting Genes) database [65]. According to the regulatory relationships between key DEMs and its targets, the regulatory networks containing key miRNAs and target mRNAs were constructed and visualized by Cytoscape software, a standard tool for integrated analysis and visualization of biological networks [66].

### 4.8. Quantitative Real-Time PCR (qPCR) Analysis

Total RNA was extracted as described before. For the reverse transcription of miRNAs, miRNA specific stem-loop primers (Supplementary Table S12) and the Prime Script RT Reagent Kit (Takara Bio, Dalian, China) were specially used. qRT-PCR was performed on the ABI PRISM 7500 Real-time PCR System (Applied Biosystems, Foster City, CA, USA) using the 2× SYBR Green Master Mix reagent (Takara Bio, Dalian, China) following the steps below: initial denaturation at 95 °C for 5 min, followed by 95 °C for 15 s and 60 °C for 45 s for 40 cycles, and one cycle of 95 °C to 65 °C. All reactions were conducted in triplicate and included negative controls with no template. The relative expression levels of the DEMs were measured in terms of threshold cycle value (*C*_t_) and were normalized to 5S rRNA using the equation 2^−ΔΔ*C*t^.

## 5. Conclusions

In conclusion, we identified that DEMs derived from intestines, livers, and brains of HSD treated *M. amblycephala* as compared to NSD treatment were related to glucose metabolic pathways and biosynthetic processes, including the GO classifications of gluconeogenesis and the glucose metabolic process, and the KEGG pathways of glycolysis/gluconeogenesis, PPAR signaling pathway, and insulin signaling pathways. Our study provides the miRNA characterization in response to high starch treatment, and these findings hint that miRNAs might play crucial roles in glucose metabolism, and different tissues (intestines, livers, and brains) confer different miRNA characterization in response to high glucose treatment. These results may be crucial for providing more information on exploring more strategies for *M. amblycephala* feeding prescription design or metabolism regulation of nutritional physiology.

## Supplementary Materials

Supplementary materials can be found at www.mdpi.com/1422-0067/18/6/1161/s1.
