# Supplementary material for: Identification of Differentially Expressed Micrornas Associate with Glucose Metabolism in Different Organs of Blunt Snout Bream (Megalobrama amblycephala)
Source: Int J Mol Sci. 2017 May 31;18(6):1161. doi: 10.3390/ijms18061161 (PMC5485985; doi:10.3390/ijms18061161)
Supplement: Supplementary file 1 [file ijms-18-01161-s001.zip › figure-S.zip!/Additional file2: Figure S1.pdf]

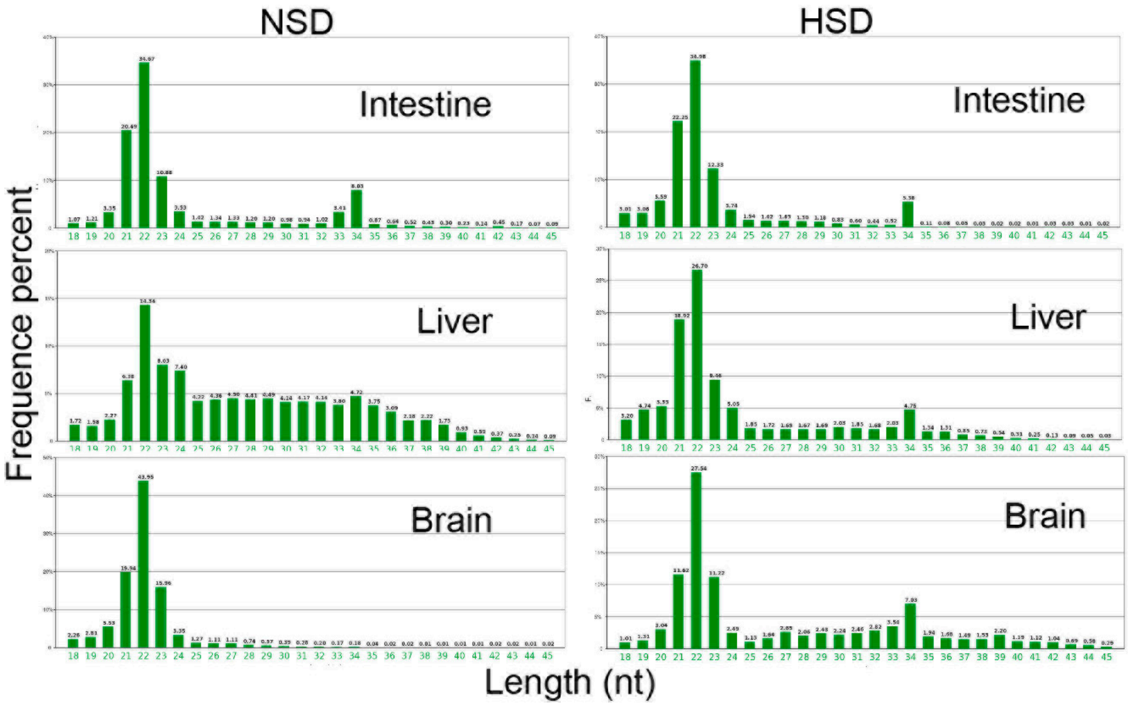

Figure S1: Length distribution of small RNAs in six libraries clean reads.
