# Supplementary material for: Identification of Differentially Expressed Micrornas Associate with Glucose Metabolism in Different Organs of Blunt Snout Bream (Megalobrama amblycephala)
Source: Int J Mol Sci. 2017 May 31;18(6):1161. doi: 10.3390/ijms18061161 (PMC5485985; doi:10.3390/ijms18061161)
Supplement: Supplementary file 1 [file ijms-18-01161-s001.zip › figure-S.zip!/Additional file3: Figure S2.pdf]

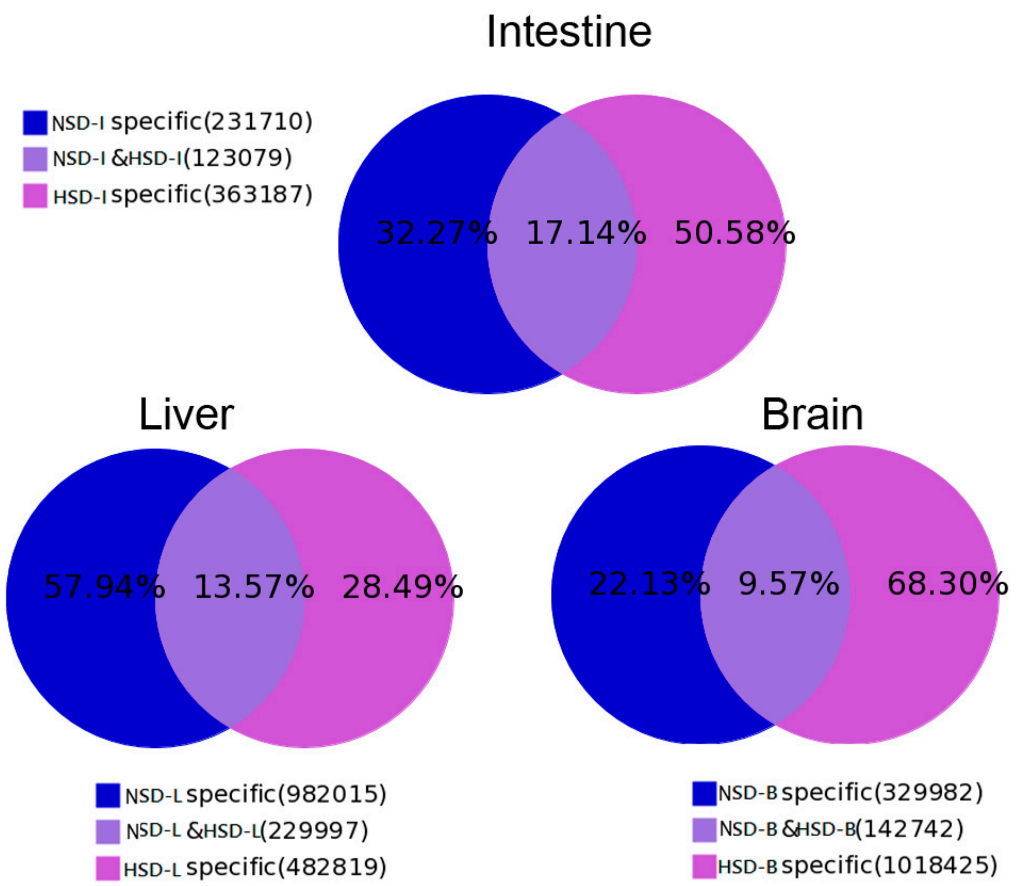

Figure S2. Venn chart for commonly and specific expressed unique small RNAs sequences in different tissues of *M. amblycephala* fed with normal (NSD) and high starch diet (HSD).
