# Supplementary material for: Identification of Differentially Expressed Micrornas Associate with Glucose Metabolism in Different Organs of Blunt Snout Bream (Megalobrama amblycephala)
Source: Int J Mol Sci. 2017 May 31;18(6):1161. doi: 10.3390/ijms18061161 (PMC5485985; doi:10.3390/ijms18061161)
Supplement: Supplementary file 1 [file ijms-18-01161-s001.zip › figure-S.zip!/Additional file5: Figure S4.pdf]

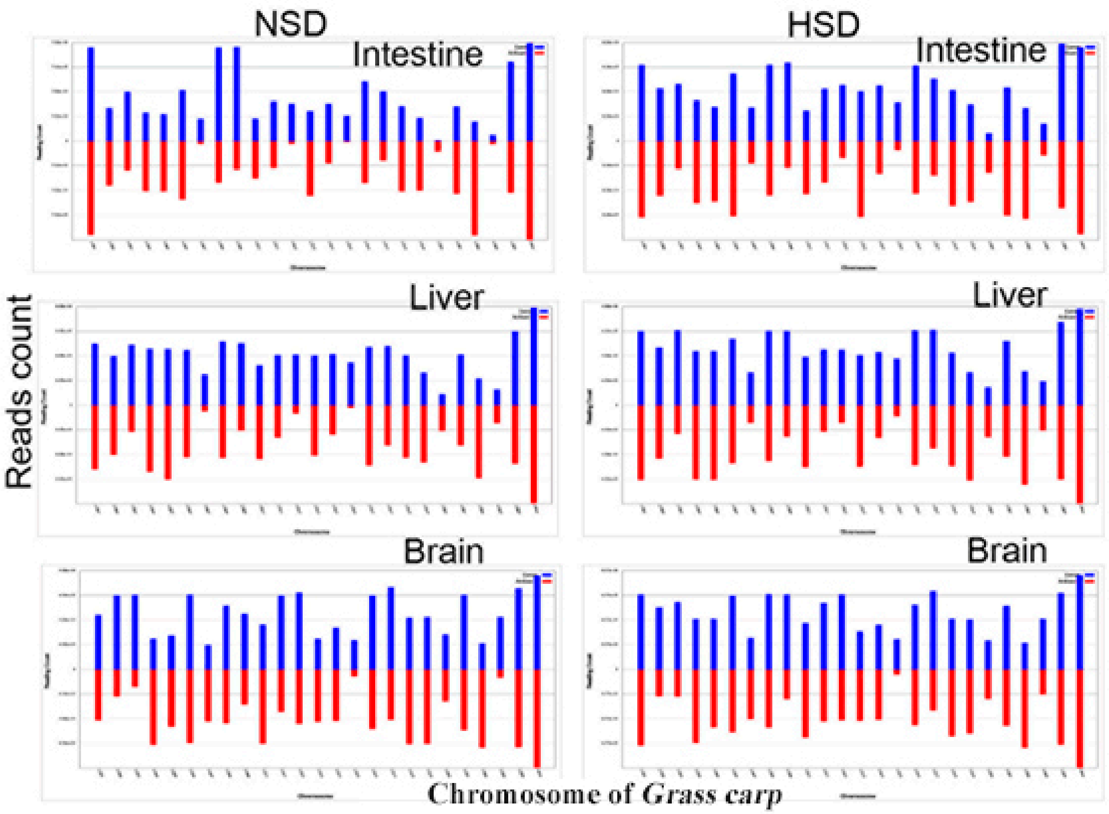

Figure S4. Number and distribution of clean reads mapped to the genome sequence of grass carp. The number of sRNAs on the sense strand of chromosome was shown in blue, whereas the number of sRNAs on the antisense strand of chromosome was shown in red.
